# Modeling Habitat Split: Landscape and Life History Traits Determine Amphibian Extinction Thresholds

**DOI:** 10.1371/journal.pone.0066806

**Published:** 2013-06-20

**Authors:** Carlos Roberto Fonseca, Renato M. Coutinho, Franciane Azevedo, Juliana M. Berbert, Gilberto Corso, Roberto A. Kraenkel

**Affiliations:** 1 Departamento de Botânica, Ecologia e Zoologia, Universidade Federal do Rio Grande do Norte, Natal, Brazil; 2 Instituto de Física Teórica, Universidade Estadual Paulista, São Paulo, Brazil; 3 Departamento de Biofísica e Farmacologia, Universidade Federal do Rio Grande do Norte, Natal, Brazil; Texas Tech University, United States of America

## Abstract

Habitat split is a major force behind the worldwide decline of amphibian populations, causing community change in richness and species composition. In fragmented landscapes, natural remnants, the terrestrial habitat of the adults, are frequently separated from streams, the aquatic habitat of the larvae. An important question is how this landscape configuration affects population levels and if it can drive species to extinction locally. Here, we put forward the first theoretical model on habitat split which is particularly concerned on how split distance – the distance between the two required habitats – affects population size and persistence in isolated fragments. Our diffusive model shows that habitat split alone is able to generate extinction thresholds. Fragments occurring between the aquatic habitat and a given critical split distance are expected to hold viable populations, while fragments located farther away are expected to be unoccupied. Species with higher reproductive success and higher diffusion rate of post-metamorphic youngs are expected to have farther critical split distances. Furthermore, the model indicates that negative effects of habitat split are poorly compensated by positive effects of fragment size. The habitat split model improves our understanding about spatially structured populations and has relevant implications for landscape design for conservation. It puts on a firm theoretical basis the relation between habitat split and the decline of amphibian populations.

## Introduction

One-third of the world’s amphibian species are threatened, more than 40% have declining populations, and 168 species probably went extinct in the last five centuries [1]. In biodiversity hotspots, 2841 amphibian species are facing an unprecedented contraction of their geographic area [2], being threatened by habitat loss and fragmentation [3]. Many theoretical models have been proposed to capture the complexity of such processes, from the theory of island biogeography [4], [5] to complex spatially explicit metapopulation models [6], [7]. The basic predictions of these models have been corroborated for different taxa, including protozoa [8], butterflies [9], [10], birds [11], and mammals [12, but see 13,14,15]. However, since amphibian species exhibit marked ontogenetic habitat shifts, being strongly affected by habitat split [16], the predictive power of such models is limited.

Habitat split is defined as human-induced disconnection between habitats used by different life history stages of a species [16]. For forest-associated amphibians with aquatic larvae, deforestation causes spatial disjunction between the habitat of the larvae, ponds and streams, and the habitat of the adults, the forest fragments. At the local scale, habitat split compels adults to traverse the anthropogenic matrix to reach breeding sites and recently metamorphosed juveniles to walk haphazardly through the matrix searching for an isolated forest fragment. This compulsory bi-directional migration causes drastic declines on amphibian populations [17].

At the landscape scale, habitat split decreases the richness of the amphibian community due to the extinction of aquatic larvae species [16]. More importantly, the richness of amphibians with aquatic larvae has been demonstrated to be more strongly affected by habitat split than by habitat loss and habitat fragmentation [16].This process causes bias in communities towards amphibians with terrestrial development, since these species are able to breed successfully in forest fragments even in the absence of a water source [16], [18].

The habitat split concept has also contributed to conservation issues. In a recent complementarity exercise for the identification of key Neotropical ecoregions for amphibian conservation, the differentiation between species with aquatic and terrestrial developmental generated a more comprehensive coverage of priority ecoregions than when species were pooled together [19]. Also, by analyzing how the incidence of habitat loss and habitat split varies across a regional landscape, the selection of a minimum priority set of watersheds for amphibian conservation could be optimized [20].

Habitat split is a worldwide phenomenon, being particularly common in biodiversity hotspots where habitat fragmentation is intense and human settlements are generally concentrated on the valleys where water is available for agriculture, industry, and human consumption [2]. The Brazilian Atlantic Forest, for instance, is now distributed in 245,173 forest fragments from which 83.4% are smaller than 50 ha [21]. In a typical Atlantic Forest landscape, less than 5% of the fragments are connected to streams [17]. In fact, “dry fragments” are the rule and their distance to the nearest stream can vary widely from a few meters to several kilometers. Predicting how the amphibian populations respond to such landscape alteration is essential for conservation.

We provide, here, the first theoretical model for habitat split. A minimum diffusion model shows that habitat split generates critical split distances for population persistence in forest fragments. The model predicts how life history traits, such as juvenile dispersal ability and recruitment, determine the extinction threshold. Furthermore, it predicts how population size is affected by the quality of the matrix and its distance from the breeding habitat. The model has relevant implications for amphibian conservation landscape design.

## Materials and Methods

### The Model

**Figure 1:**
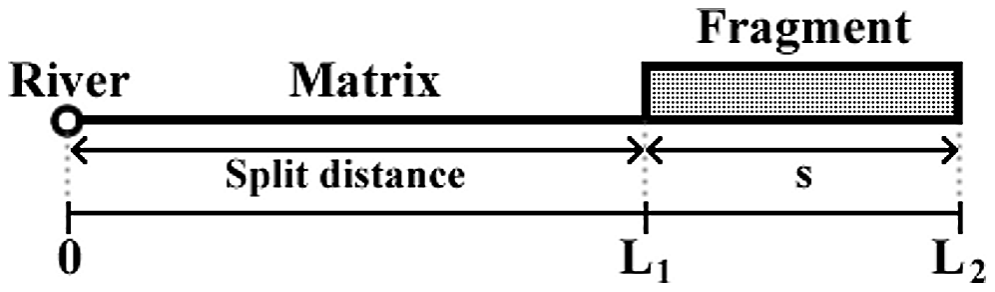
The spatial configuration for the habitat split model. The model has three main landscape elements: the river (or any aquatic breeding habitat), at 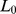, the inhospitable matrix and the forest fragment. Split distance is defined by 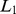 while fragment size (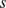) is defined by 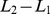.

In this section we develop a model designed to capture the main consequences of habitat split on populations of amphibians with aquatic larvae. This means a model that has enough ingredients to provide a basis for predictions without, however, taking into account particularities of any specific amphibian species. The main point the model is set to address is that of population decline and local extinction. The spatial configuration of the model is shown in Figure 1. The reproductive site of the amphibians is at the river at 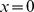, whereas the forest fragment, where the adults live, has a size 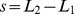. The shortest distance from the fragment to the river is 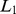, from now on called *split distance*, a new metric for habitat fragmentation studies. We have chosen to work in a one-dimensional context. Extensions to a two-dimensional space can be implemented, but the main features are already present in our model.

Habitat split consequences on the amphibian population are directly connected to the fact that the population is stage-structured. Accordingly, we introduce two variables, 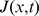 and 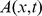, which represent juveniles and adults densities, respectively. We will assume that after leaving the reproductive site the juvenile amphibians move in a haphazard way through the matrix. This assumption is based on the fact that in the pristine environment they did not have to search for the terrestrial habitat, therefore they lack adaptations that allow them to find the forest fragments in a directional fashion. From the modeling point of view, this suggests that a diffusion equation is appropriate to describe the spatial aspects of the juveniles in the matrix. In the fragment, we will posit that the adults also obey a diffusion equation, assuming that the forest fragment is spatially homogeneous and that the adults will haphazardly look for food and escape from their predators before reproduction. However, individuals walking in the matrix or in the forest fragment can exhibit different diffusion rates.

Juveniles that reach the fragment are dynamically equivalent to adults, so we will assume that there are no juveniles in the fragment, 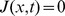 when 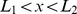. On the other hand, adults migrate through the matrix back to the river for reproduction. This however, is a directed movement, much more like advection rather than diffusion, and is not modeled explicitly. Mathematically, these assumptions translate into two diffusive equations. The first equation is defined for juveniles in the matrix and the second for adults in the forest fragment:
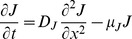
(1)

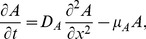
(2)Where 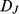 and 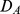 are the diffusion coefficients for juveniles and adults in the matrix and the forest, and 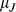 and 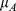 the respective mortality rates. At the fragment border 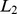, several scenarios are possible, depending on the landscape beyond 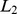: the boundary may be completely absorbing if there is a very hostile matrix, or totally reflexive if the environment is as good as in the fragment, or it can be something in between. This point will be discussed in detail in the next section and for now we consider a general formulation [22]:



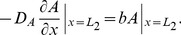
(3)If 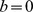, we have a completely closed patch at 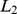 and the adults will turn back towards the fragment interior. This condition is used when we do not want to take into account size effects of habitat patch, that is, when the patch is large. The opposite limit, 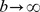, corresponds to the situation in which all individuals that reach the border 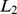 will leave the modeled landscape.

When juveniles reach the fragment, they become adults and, since adults cannot turn into juveniles, the border 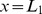 represents a completely absorbing boundary for juveniles. Moreover, the rate at which new adults arrive at the fragment must be the same as the rate of juveniles leaving the matrix. These conditions are expressed in the following boundary conditions:
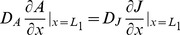
(4)

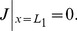
(5)


The fourth and last boundary condition models the reproductive behavior of the amphibians. For simplicity, adults are assumed to exhibit a constant recruitment rate 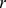, so that the rate at which new juveniles are generated at the river is proportional to the total number of adults in the fragment. We also take into account that it takes a certain time, 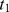, for the influx of juveniles to respond to a variation in the number of adults. Population size is controlled by competition at the river, so we introduce a saturation parameter 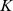, which can be interpreted as the maximum rate of juveniles that can be generated. The mathematical expression of this condition is the following:
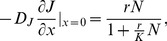
(6)where 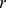 is the recruitment rate and 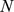 is the total number of adults in the fragment at a past time. Notice that the negative sign accounts for the fact that 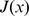 is always a decreasing function of 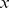. The total number of adults in the fragment at a past time is given by:



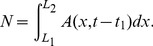
(7)In this model we suppose that the most important factor limiting amphibian flow is of the juveniles that start at the river and cross the matrix to the forest fragment. The return of the adults to the river is assumed in equation (6) to be advective. These conditions introduce two phenomenological constants 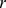 and 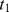. The first of them, the recruitment, takes into account the fertility of adults, the survival of tadpoles and the adult mortality in the matrix. The time 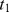 is the sum of the time to cross the matrix, mate, reproduce, mature eggs and develop juveniles capable of crossing the matrix. Although describing a different system, this set of equations and boundary conditions is similar to the one presented in [23].

## Results

Equations (1,2) do not contain any density dependent terms: they are linear. As discussed above, the population control term appears only in the boundary conditions, namely in equation (6). Moreover, the fact that these conditions include a time delay makes it impossible to obtain exact solutions in general. However, when we seek for stationary solutions, that is, solutions such that 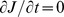 and 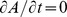, the time delay plays no role anymore and we can find the solutions and – more important – the existence criteria for non-zero solutions.

The stationary solutions are obtained by setting to zero the time-derivatives in equations (1,2) which leads us to:
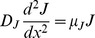
(8)

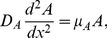
(9)where we have changed partial derivatives for ordinary ones as 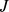 and 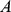 depend only on 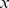.The couple of linear equations (8,9) has the solution:



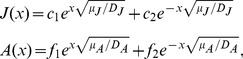
(10)Where 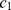, 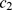, 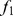 and 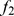 are integration constants. With help of boundary conditions (5,6) we find explicitly their values. This result is found in the supplementary material S1. Equation (10) makes sense only for real positive solutions. We derive such conditions from 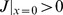. If this condition is not satisfied the population will go extinct as the null solution turns out to be the only stable one in this case. We prove in supplementary material S1 that 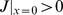 is equivalent to:



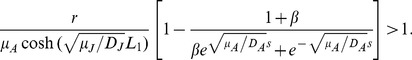
(11)In this expression, 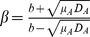. This condition has a standard interpretation in population dynamics: the recruitment should be large enough to replace the population, otherwise, the population disappears. In the special case where we take to be zero, we have:
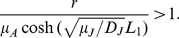
(12)


Further, notice that 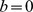 is the same as taking the limit 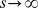, that is, considering an arbitrarily large patch.

### Stationary Solutions

**Figure 2:**
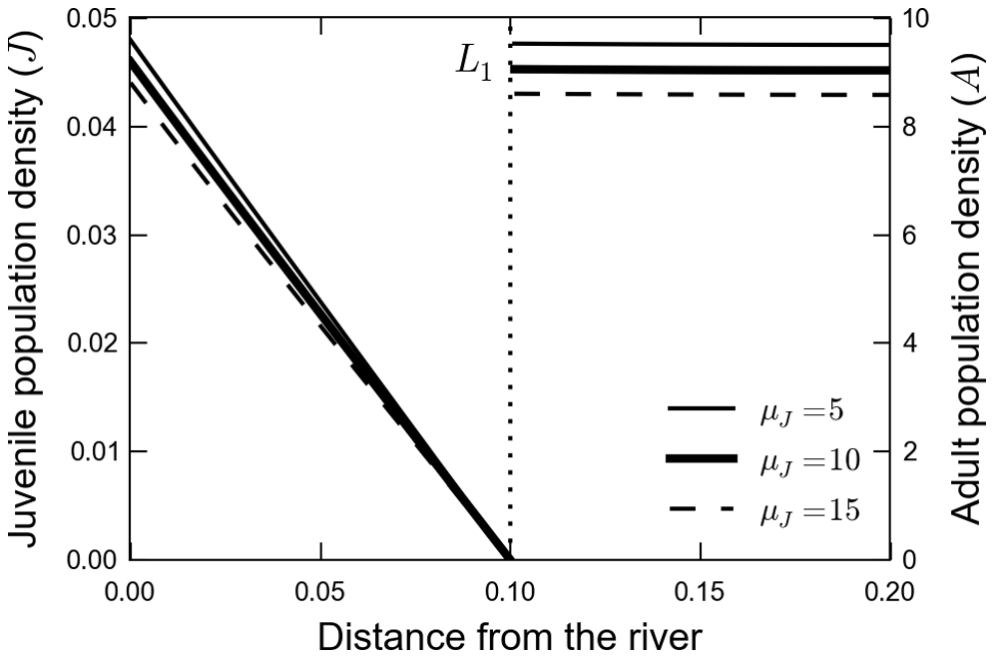
Stationary solutions of the model as a function of space. In the model, all distances are measured according to the reproductive site that is taken as zero. The fragment starts at 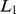 and ends at 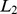. Each curve refers to a given juvenile mortality in the matrix (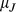). The parameters used were 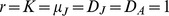, 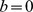 and 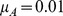.

The general form of the stationary solution as a function of 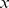, the distance from the river, is depicted in Fig. 2, where we assume now 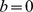. To help the visualization we plot the juveniles and adults in the same figure; for 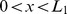 the density in the y-axis refers to juveniles while for 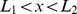 the density of the adults is plotted. As a first approach we assume that diffusion coefficients of adults and juveniles are the same: 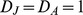. On the other hand we suppose a large difference in mortalities of juveniles and adults, we use 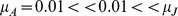. The values of 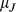 are shown in the picture. In this and the following plot we use 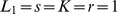. The general behavior of this solution points to populations that decrease in the matrix and tend to stabilize in the fragment.

We also explore in Fig. 2 the dependence of the population in the fragment on juvenile mortality. We plot solutions for three distinct 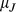, simulating matrixes of different quality. As expected, an increase in the mortality leads to smaller populations and, as a preview of the next subsection, this trend suggests the existence of a threshold in this model.

### The Existence of a Critical Split Distance

The split distance, 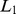, is an important landscape metric which has great influence on the existence of a non-zero stationary solution of the model and therefore on the viability of the population. At this point we explore the most important conceptual result of this work. The model introduced in this article predicts an extinction threshold for 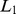. This means, there is a critical split distance 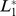 such that if 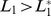 the amphibian population goes locally extinct. In other words, if the split distance is larger than a certain value, the population does not persist in this landscape.

**Figure 3:**
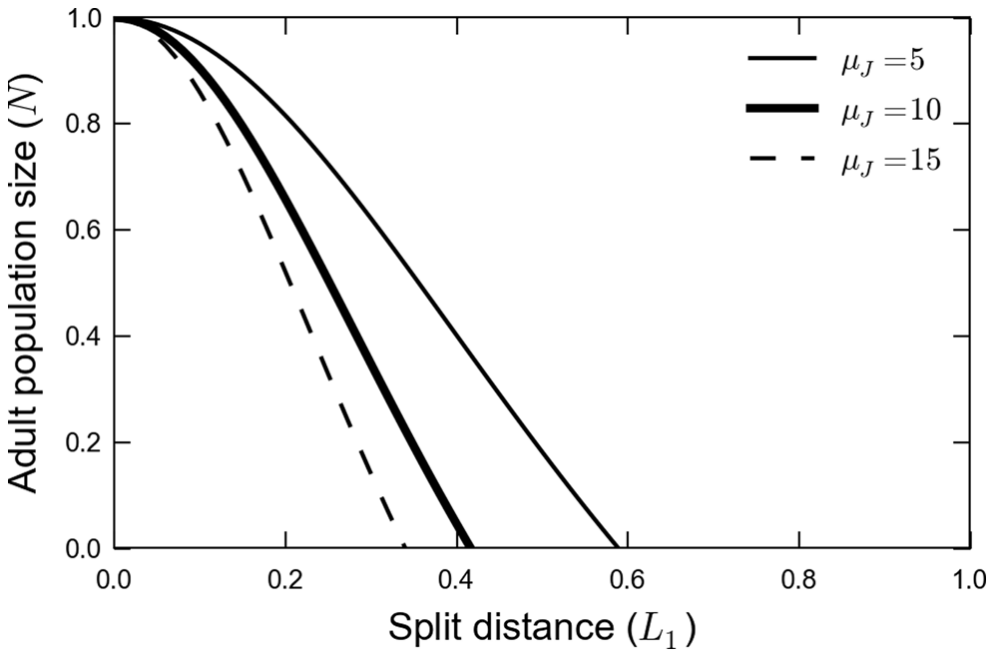
Population size of adults in the fragment as a function of the split distance, 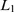
 This picture points a critical split distance 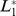 above which the population gets extinct. The three curves indicated in the figure represent different values of the juvenile mortality 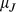 that can be caused by differences in matrix quality. The parameters used were 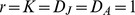, 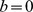 and 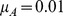.

In Fig. 3 we show the population size in the fragment as a function of the split distance 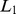, for three different values of the juvenile mortality 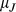. The presence of a critical value 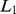 (the point where the curves intercept the x-axis) is clearly seen. This figure also explores the influence of 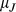 on 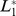; as expected there is an inverse relation between 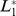 and 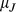. A more inhospitable habitat (large 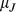) will make the critical split distance smaller.

### Dependence of the Critical Split Distance on Life-history Parameters

**Figure 4:**
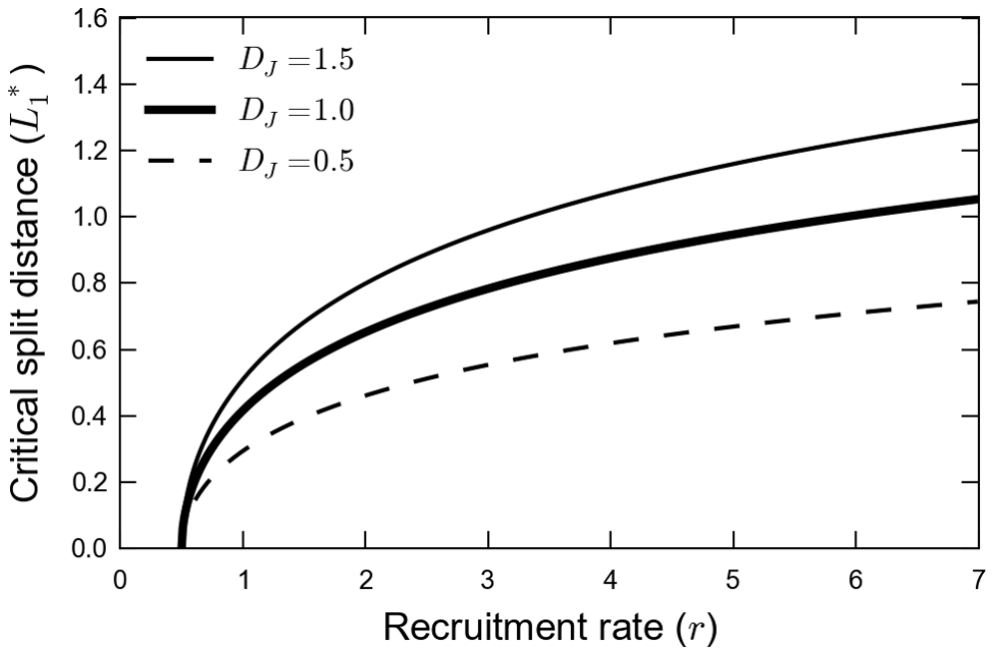
Critical split distance as a function of life history traits. The model explores two factors that modulate the habitat split effect: the recruitment rate and the diffusion coefficient of the juveniles. The parameters used were 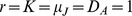, 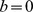 and 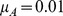.

One of the most relevant life-history traits for our analysis is the recruitment 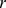, a parameter that measures the reproductive success of the amphibians. Indeed, in our model 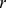 encompasses three biological processes: fertility of the reproductive adults, the survival of the tadpoles until they emerge from the aquatic habitat to become able to cross the matrix and the adult mortality in their way back to the river to reproduce. In Fig. 4 we show the critical split distance, 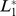, as a function of the recruitment, 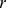, for three distinct values of diffusion coefficients of the juveniles 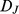. The point (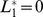, 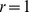) is a limit case; for this situation the recruitment is the minimum to maintain the population (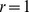) when the favorable habitat is connected to the reproduction site (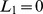). The curves of Fig. 4 show an increase of 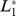 with recruitment translating the fact that a higher reproductive success allows for larger split distances. The reason of this behavior is that the recruitment compensates the mortality in the matrix.

The three curves in Fig. 4 examine the influence of the diffusion of the juveniles, 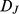; for a given 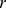, a larger diffusion coefficient allows a larger split distance for the population. In this way 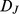
also counterbalances the mortality in the matrix: higher dispersal ability helps to deplete the effect of habitat split.

### Dependence of the Critical Split Distance on Landscape Metrics

**Figure 5:**
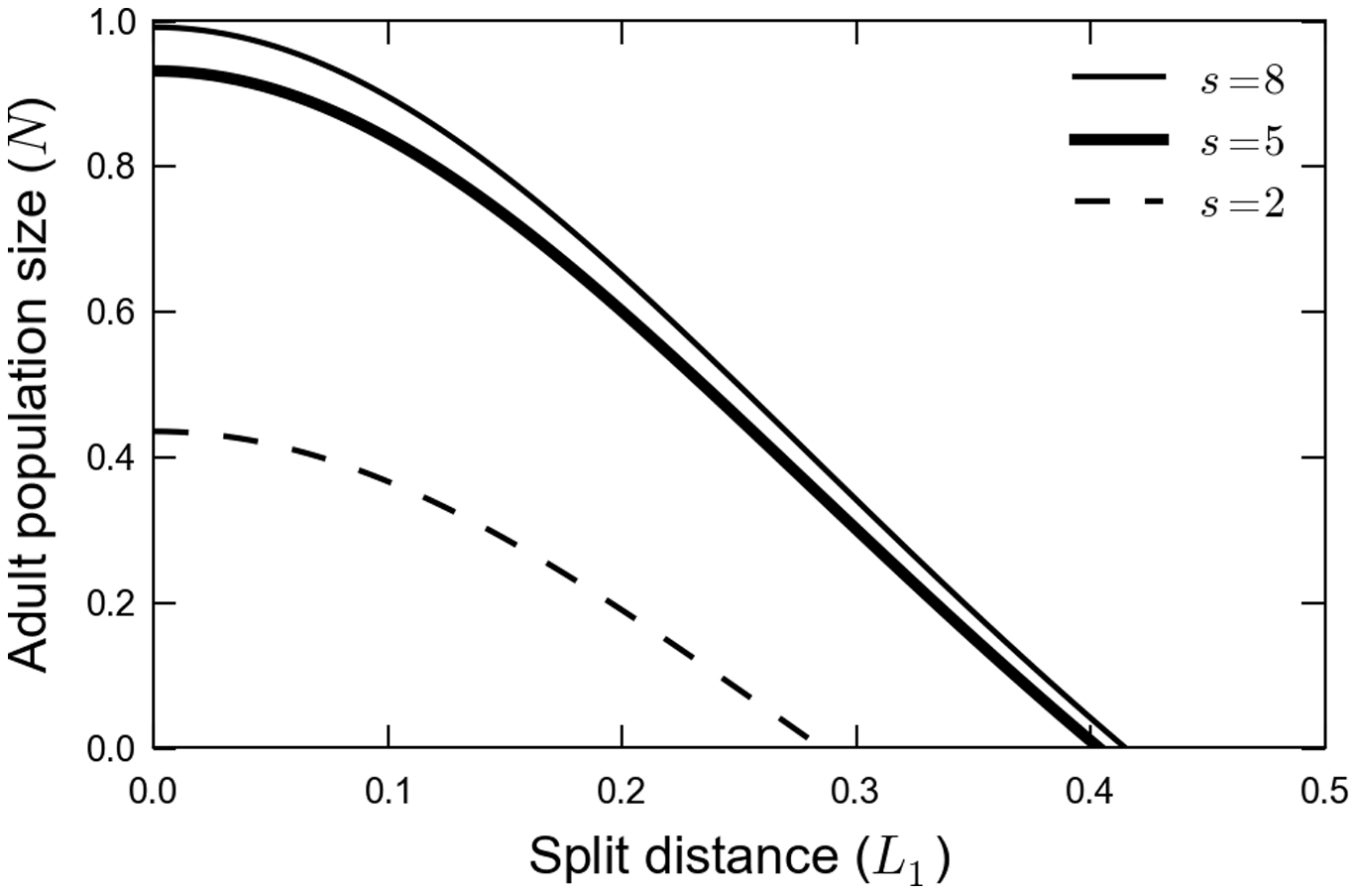
Curve of adult population size versus split distance. In this figure we point the effect of the size of the fragment on the critical split distance. This figures show that large forest fragments have only a limited effect to reduce the habitat split local extinction prevision. The parameters used 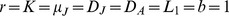 and 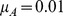.

As we have seen in the previous sections, a critical split distance appears. When we took the border of the fragment as completely reflexive (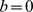), the dependence of the critical split distance on the size of the fragment disappeared: no matter how large the fragment, once a critical split distance is attained, the population goes locally extinct. On the other hand, we can introduce a non-zero value for 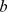, representing a partially absorbing boundary at 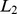. In this case, a flux of adults to the outside of the fragment exists, making it still more difficult for the population to persist. To illustrate this point, we plotted in Fig. 5 the adult population in the fragment as a function of the split distance for three different fragment sizes in the case where 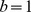. It is clear that the population is always smaller the smaller the fragment is, representing a typical area effect.

## Discussion

Amphibian populations are declining worldwide [24], [25]. Several non-exclusive hypotheses have been proposed to explain such a widespread pattern, including the emergence of *Batrachochytrium dendrobatidis*, a highly virulent fungus [26], [27], ultraviolet-B radiation, climate change [28], pollution [29], introduction of exotic species [30], habitat loss and fragmentation [1], [31], [32], and, more recently, habitat split [16]. Here, we explore the theoretical consequences of habitat split for the conservation of amphibian species with aquatic larvae. However, the model can be of relevance for other organisms exhibiting marked ontogenetic habitat shifts. For instance, insects with indirect development, such as dragonflies and damselflies, have been recently demonstrated to suffer from alterations in the physical structure of the riparian vegetation that disconnect the aquatic habitat of the larvae from the terrestrial habitat of the adults [33].

Our diffusive model reveals that habitat split alone can generate extinction thresholds. Fragments located between the breeding site and a given critical split distance are expected to contain viable amphibian populations. In contrast, populations inhabiting fragments farther from such critical distance are expected to be extinct. The theoretical existence of extinction thresholds has been also demonstrated for habitat loss and fragmentation [6], [34], [35]. In this case, increase in the proportion of habitat loss above a certain level causes an abrupt non-linear decay in population size. Simulation models based on percolation theory suggest that this can be simply attributed to structural properties of the fragmentation process [36]. However, biological mechanisms such as minimal home range, minimal population size, and the Allee effect contribute to such extinction thresholds [35].

The model can be also interpreted from a breeding site perspective. Split distance is expected to have a negative impact on the occupancy of matrix-inserted ponds. Indeed, a field study with *Rana dalmatina* demonstrated that the number of egg-clutches in ponds declines exponentially with increasing distance from a deciduous forest. Ponds less than 200 m from the forest edge were considered the most valuable for the species conservation [37]. For *Rana temporaria*, the occupancy of ponds for breeding purposes is influenced by the distance from suitable summer habitats [38]. Furthermore, some studies have shown that the richness of amphibians in ponds is negatively related to the distance to forest patches [39], [40].

The habitat split model predicts that amphibian species with different life history traits will exhibit different extinction proneness in response to a given landscape setting. One key feature determining the critical split distance (i.e. the distance value of 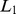 at which the predicted abundance is zero) is the diffusion rate of the post-metamorphic juveniles in the matrix. Amphibian species with higher diffusion rate are expected to exhibit farther extinction thresholds. Therefore, for a given fragmented landscape submitted to habitat split, species with lower diffusion ability are expected to be present in a smaller number of fragments and ultimately be regionally extinct earlier. Additionally, the probability of extinction in response to habitat split gradients can be expected to be higher for species with lower diffusion ability.

Amphibians vary considerably in dispersal ability. Across species, the frequency distribution of maximum dispersal distance fits an inverse power law [41].While 56% of the amphibian species presented maximum dispersal distances lower than one kilometer, 7% could disperse more than 10 km [41]. However, those are data for adults. For the habitat split model, the main parameter to be estimated is the diffusion rate of the post-metamorphic juveniles. Although such data is more difficult to be obtained, one expects that the mean dispersal ability of juveniles should be lower than of adults due to their smaller body size, lower energetic reserves and higher sensitivity to environmental stress [42].

The reproductive success is also a crucial life history parameter determining how far is the critical split distance for a given species. In the model, reproductive success is defined as the average number of post-metamorphic juveniles produced per adult living in the fragment. Since this parameter varies between species, we expect that species with higher reproductive success will be able to keep viable populations in forest fragments that are farther away from the breeding site in comparison to species with lower reproductive success. We envisage that in future individual-based models, recruitment could vary between individuals according to their conditions.

Reproductive success is positively correlated to clutch size but also a function of the survival rate of the aquatic larvae before metamorphosis. Body size is possibly a good inter-specific predictor of reproductive success. Body size has a strong positive inter-specific relationship with clutch size, even after controlling for the phylogeny [43]. Furthermore, for pond-breeding anurans of three different families (Bufonidae, Hylidae and Ranidae), there is a positive relationship between body size and egg-diameter [42]. Therefore, species with larger body size can be expected to exhibit larger critical split distances. The survival rate of the aquatic larvae, however, depends on biotic interactions, such as predation and competition. Furthermore, riverine systems are nowadays submitted to multiple anthropogenic generated stressors [44], agrochemicals [29], and emerging diseases [26] that can dramatically alter mortality rates.

The model predicts that the extinction threshold can be pushed away from the breeding site only at the cost of increasing the size of the fragment, depending on the kind of boundary at the interface habitat/exterior matrix. However, although enlarging the size of the fragment can allow for larger splits, even in the case of a very large fragment the critical split distance remains finite: local extinction can occur even for infinite fragments when the split distance is larger than a critical value. The implications of such results for conservation are straightforward. When habitat loss is intense and small fragments are the rule, the best landscape scenario for the conservation of forest-associated amphibians with aquatic larvae is the preservation of the riparian vegetation.

The quality of the matrix is also a key element defining the critical split distance. Higher quality matrix generates lower mortality rates of post-metamorphic juveniles enabling recruited individuals to successfully reach forest fragments that are farther away. For empirical studies this parameter is critical since anthropogenic matrix vary widely in quality, from intensively used cattle and crop fields to ecologically-managed tree monocultures [45]. Furthermore, roads are also important matrix elements that can jeopardize the bi-directional migration of amphibians [46].

In biodiversity hotspots [2], in particular, landscape design is expected to play a crucial role in the conservation of the aquatic larvae species. For instance, the Brazilian Atlantic Forest is home of one the most species rich amphibian fauna of the world [2], containing at least 300 endemic amphibian species [47]. Nowadays, only 11.7% of its original cover is left, and although the protection of the riparian vegetation was, until recently, insured by the Brazilian Forest Code (4771/65), habitat split is a common feature in the landscape [16]. Not surprisingly, several amphibian populations have declined recently [48], [49], [50] and many more are expected to pay the extinction debt [51]. The habitat split model reinforces the view that the conservation and restoration of riparian vegetation should be properly enforced [52].

Metapopulation models assume disjunct breeding patches containing individual populations that exist in a shifting balance between extinctions and recolonisations via dispersing individuals [6]. Realistic models on metapopulations have incorporated patch area, shape, isolation besides the quality of the intervening matrix [7]. The metapopulation concept has been applied to amphibians, showing even structured genetic outcomes [53]. Despite that, the application of metapopulation models to amphibians has been questioned in several grounds [14], [41]. We envisage that future metapopulation models, when designed to species exhibiting marked ontogenetic habitat shifts, will generate more accurate predictions by the incorporation of habitat split effects.

## Supporting Information

File S1 **Mathematical formulation of the model, including the derivation of the stationary solution and a study of its stability.**
(PDF)
