# Supplementary material for: Modeling Habitat Split: Landscape and Life History Traits Determine Amphibian Extinction Thresholds
Source: PLoS One. 2013 Jun 20;8(6):e66806. doi: 10.1371/journal.pone.0066806 (PMC3688573; doi:10.1371/journal.pone.0066806)
Supplement: File S1 — Mathematical formulation of the model, including the derivation of the stationary solution and a study of its stability. (PDF) [file pone.0066806.s001.pdf]

Supporting Information Material S1

Mathematical formulation of the model

We define again the equations for juveniles in the matrix, J, and for adults in the forest fragment, A:

$$\frac{\partial J}{\partial t} = D_J \frac{\partial^2 J}{\partial x^2} - \mu_J J \tag{S.1}$$

$$\frac{\partial A}{\partial t} = D_A \frac{\partial^2 A}{\partial x^2} - \mu_A A \ , \tag{S.2}$$

where D_J and D_A are the diffusion coefficients for juveniles in the matrix and for adults inside the forest, and μ_J and μ_A the respective mortality rates. For this system, we define boundary conditions considering that juveniles become adults when they reach the fragment (Eqs. (S.3) and (S.4)). Since adults do not become juveniles, that border is completely absorbing to the juvenile population:

$$J\big|_{x=L_1} = 0 \ . \tag{S.3}$$

We must also observe that the flux of adults that enter the fragment must be the same as the flux of juveniles leaving the matrix, so that

$$D_A \frac{\partial A}{\partial x}\bigg|_{x=L_1} = D_J \frac{\partial J}{\partial x}\bigg|_{x=L_1} \ . \tag{S.4}$$

Further, at the fragment border, several scenarios are possible according to the landscape beyond: (i) the boundary may be completely absorbing, if there is a very hostile matrix for $x > L_2$, or (ii) totally reflexive, if the environment is as good as in the fragment, or (iii) it can be something in between. This point yields the third condition:

$$-D_A \frac{\partial A}{\partial x}\bigg|_{x=L_2} = bA\bigg|_{x=L_2} \ , \tag{S.5}$$

where b defines how reflexive or absorbing the border at $x = L_2$ is. When $b = 0$ means that the patch is large, and it is used to avoid size effects of habitat patch.

The fourth boundary condition deals with the reproductive behavior of the amphibians. The population of juveniles generated at the river at time t is proportional to the total number of adults in the fragment at an earlier time $t - t_1$. To control the population size we include a saturation parameter K, that considers the intraspecific competition at the river. Then, we have:

$$-D_J \frac{\partial J}{\partial x}\bigg|_{x=0} = \frac{rN}{1 + \frac{r}{K}N} , \tag{S.6}$$

where r is the recruitment of new individuals and N is the total adult population at the fragment in a previous time t_1 given by:

$$N = \int_{L_1}^{L_2} A(x, t - t_1) dx . \tag{S.7}$$

This model has the diffusive behavior of juveniles crossing the matrix searching for the fragment and the advective movement of adults during their return to the river to mate and reproduce. The condition (S.6) introduces two important phenomenological constants r and t_1. The first is the recruitment depending on the fertility of adults, the survival of tadpoles and the adult mortality in the matrix. The second, t_1, is the sum of the time expent by adults to cross the matrix, mate, reproduce, plus the time to mature eggs and develop juveniles capable of crossing the matrix.

Derivation of the stationary solution

The model equations (1 and 2), along with the boundary conditions (3, 4, 5 and 6), are studied qualitatively through the analysis of fixed points, namely, through stationary solutions, which do not depend on time, thus $A(x,t) = A^*(x)$ and $J(x,t) = J^*(x)$. In this case, we have that $\partial J/\partial t = 0$ and $\partial A/\partial t = 0$, leading to equations (8 and 9):

$$D_J \frac{d^2 J}{dx^2} = \mu_J J$$
$$D_A \frac{d^2 A}{dx^2} = \mu_A A .$$

The solution yields equation (10):

$$\begin{cases} J(x) & = c_1 e^{\sqrt{\frac{\mu_J}{D_J}}x} + c_2 e^{-\sqrt{\frac{\mu_J}{D_J}}x} \\ A(x) & = f_1 e^{\sqrt{\frac{\mu_A}{D_A}}x} + f_2 e^{-\sqrt{\frac{\mu_A}{D_A}}x} , \end{cases}$$

where the constants c_1, c_2, f_1 and f_2 from integration are fixed by the boundary conditions, that do not depend on time:

$$J\Big|_{x=L_1} = 0 \tag{S.8}$$

$$D_J \frac{\partial J}{\partial x}\Big|_{x=L_1} = D_A \frac{\partial A}{\partial x}\Big|_{x=L_1} \tag{S.9}$$

$$-D_A \frac{\partial A}{\partial x}\Big|_{x=L_2} = bA\Big|_{x=L_2} , \tag{S.10}$$

$$-D_J \frac{\partial J}{\partial x}\Big|_{x=0} = \frac{rN}{1+\frac{r}{K}N} \tag{S.11}$$

Using the boundary condition (S.10), we find

$$f_2 = f_1 e^{2\sqrt{\frac{\mu_A}{D_A}}L_2} \left(\frac{\sqrt{\mu_A D_A}+b}{\sqrt{\mu_A D_A}-b} \right) , \tag{S.12}$$

with $\beta = (b+\sqrt{\mu_A D_A})/(b-\sqrt{\mu_A D_A})$, we rewrite :

$$f_2 = -f_1 e^{2\sqrt{\frac{\mu_A}{D_A}}L_2} \beta , \tag{S.13}$$

From equation (S.8)

$$c_1 = -c_2 e^{-2\sqrt{\frac{\mu_J}{D_J}}L_1} ,$$

then, from equation (S.9), we obtain c_1 and c_2 as functions of f_1:

$$c_1 = f_1 \frac{\sqrt{\mu_A D_A}}{\sqrt{\mu_J D_J}} \frac{e^{-\sqrt{\frac{\mu_J}{D_J}}L_1} e^{\sqrt{\frac{\mu_A}{D_A}}L_2} \left(e^{-\sqrt{\frac{\mu_A}{D_A}}s} + \beta e^{\sqrt{\frac{\mu_A}{D_A}}s} \right)}{2} \tag{S.14}$$

$$c_2 = -f_1 \frac{\sqrt{\mu_A D_A}}{\sqrt{\mu_J D_J}} \frac{e^{\sqrt{\frac{\mu_J}{D_J}}L_1} e^{\sqrt{\frac{\mu_A}{D_A}}L_2} \left(e^{-\sqrt{\frac{\mu_A}{D_A}}s} + \beta e^{\sqrt{\frac{\mu_A}{D_A}}s} \right)}{2} , \tag{S.15}$$

where $s = L_2 - L_1$ is the fragment size.

Calculating N as function of f_1:

$$\begin{aligned} N &= \int_{L_1}^{L_2} A(x)\, dx = \int_{L_1}^{L_2} f_1 e^{\sqrt{\frac{\mu_A}{D_A}}L_2} \left[e^{-\sqrt{\frac{\mu_A}{D_A}}(L_2-x)} - \beta e^{\sqrt{\frac{\mu_A}{D_A}}(L_2-x)} \right] dx \\ &= f_1 \sqrt{\frac{D_A}{\mu_A}} e^{\sqrt{\frac{\mu_A}{D_A}}L_2} \left(1 - e^{-\sqrt{\frac{\mu_A}{D_A}}s} + \beta - \beta e^{\sqrt{\frac{\mu_A}{D_A}}s} \right) , \end{aligned} \tag{S.16}$$

then, using the condition (S.11):

$$f_1 = \frac{K}{\sqrt{\mu_A D_A} e^{\sqrt{\frac{\mu_A}{D_A}} L_2}} \times \left\{ \frac{\mu_A}{r} \left[\beta \left(e^{\sqrt{\frac{\mu_A}{D_A}} s} - 1 \right) + e^{-\sqrt{\frac{\mu_A}{D_A}} s} - 1 \right]^{-1} - \left[\cosh\left(\sqrt{\frac{\mu_J}{D_J}} L_1 \right) \left(\beta e^{\sqrt{\frac{\mu_A}{D_A}} s} + e^{-\sqrt{\frac{\mu_A}{D_A}} s} \right) \right]^{-1} \right\} . \quad \text{(S.17)}$$

Substituting Eq. (S.17) into Eqs. (S.13), (S.14) and (S.15), all integration constants are specified as function of the model parameters. Consequently, one is able to write the stationary solution $J^*(x)$ and $A^*(x)$ in terms of the model parameters.

Existence and stability of the stationary solution

From the biological perspective, the stationary solutions shown in the previous section are significant only if their values are positive for all x. It means that $J^*(x) > 0$ and $A^*(x) > 0$. Since it can be shown that the condition for positive stationary solution at any x yields the same results, we consider the particular case $x = 0$, to derive conditions for our model to satisfy:

$$J^*(0) > 0 . \quad \text{(S.18)}$$

Thus, taking $x = 0$ in equation (10) leads to

$$c_1 + c_2 > 0 , \quad \text{(S.19)}$$

which is satisfied if, and only if

$$\frac{r}{\mu_A \cosh\left(\sqrt{\frac{\mu_J}{D_J}} L_1 \right)} \left[1 - \frac{1+\beta}{\beta e^{\sqrt{\frac{\mu_A}{D_A}} s} + e^{-\sqrt{\frac{\mu_A}{D_A}} s}} \right] > 1 . \quad \text{(S.20)}$$

In the case where the fragment border at $x = L_2$ is completely absorbing, one has $b = 0$ and $\beta = -1$, so the condition (S.20) for population persistence becomes:

$$r > \mu_A \cosh\left(\sqrt{\frac{\mu_J}{D_J}} L_1 \right) , \quad \text{(S.21)}$$

One can easily see that as $L_1 \to 0$, $\cosh\left(\sqrt{\frac{\mu_J}{D_J}} L_1 \right) \to 1$, it shows immediately that in the case where there is no split, the population persistence is possible if r satisfies

$$r > \mu_A , \quad \text{(S.22)}$$

which is a known result for population dynamics.

Further, the stability around the trivial solution $J = A = 0$ turns out to be unstable, once the stationary solution becomes positive and stable. Therefore, the conditions for stability (S.20) and (S.21) found above are also valid to analyse this particular case.
